# Cost-Effectiveness of Public Health Measures to Control COVID-19 in China: A Microsimulation Modeling Study

**DOI:** 10.3389/fpubh.2021.726690

**Published:** 2022-01-04

**Authors:** Qiang Wang, Naiyang Shi, Jinxin Huang, Liuqing Yang, Tingting Cui, Jing Ai, Hong Ji, Ke Xu, Tauseef Ahmad, Changjun Bao, Hui Jin

**Affiliations:** ^1^Department of Epidemiology and Health Statistics, School of Public Health, Southeast University, Nanjing, China; ^2^Key Laboratory of Environmental Medicine Engineering, Ministry of Education, School of Public Health, Southeast University, Nanjing, China; ^3^Jiangsu Provincial Center for Disease Control and Prevention, Nanjing, China

**Keywords:** COVID-19, cost-effectiveness, agent-based model, public health measures, China

## Abstract

This study aimed to assess the cost-effectiveness of various public health measures in dealing with coronavirus disease 2019 (COVID-19) in China. A stochastic agent-based model was used to simulate the progress of the COVID-19 outbreak in scenario I (imported one case) and scenario II (imported four cases) with a series of public health measures. The main outcomes included the avoided infections and incremental cost-effectiveness ratios (ICERs). Sensitivity analyses were performed to assess uncertainty. The results indicated that isolation-and-quarantine averted the COVID-19 outbreak at the lowest ICERs. The joint strategy of personal protection and isolation-and-quarantine averted one more case than only isolation-and-quarantine with additional costs. The effectiveness of isolation-and-quarantine decreased with lowering quarantine probability and increasing delay time. The strategy that included community containment would be cost-effective when the number of imported cases was >65, or the delay time of the quarantine was more than 5 days, or the quarantine probability was below 25%, based on current assumptions. In conclusion, isolation-and-quarantine was the most cost-effective intervention. However, personal protection combined with isolation-and-quarantine was the optimal strategy for averting more cases. The community containment could be more cost-effective as the efficiency of isolation-and-quarantine drops and the imported cases increases.

## Introduction

The global number of reported cases of coronavirus disease 2019 (COVID-19) has surpassed 256 million in more than 200 countries as of November 23, 2021 (1). The 21st century has witnessed several large-scale outbreaks of infectious diseases caused by coronaviruses. However, the number of COVID-19 cases is significantly higher than cases of severe acute respiratory syndrome (SARS) and Middle East respiratory syndrome (MERS). Statistics show that SARS caused more than 8,000 morbidities and MERS more than 2,200 morbidities in over 25 countries (2).

To prevent person-to-person transmission of COVID-19, several measurements have been implemented globally, including non-pharmaceutical interventions (NPIs) and pharmaceutical interventions (PIs). However, given humans' limited immunity, NPIs are necessary in the fight against COVID-19. The isolation of infected cases and quarantine of humans exposed to these cases were the most common public health measures. From an individual perspective, susceptible humans needed to wear masks and maintain good hygiene practices; from a national perspective, authorities introduced restrictions on public gatherings, movement, and public transportation.

The decline in the number of COVID-19-infected cases in many countries has demonstrated that NPIs are successful in preventing COVID-19 transmission. However, these measurements also bear a heavy economic burden. A previous study estimated that the cost of suppression policies might have been between US$ 632 billion and US$ 765 billion during the first wave of COVID-19 in the United States (3). The cumulative loss in the global gross domestic product (GDP) in 2020 and 2021 is estimated at ~US$ 9 trillion (4). Generally, strategies need to be developed based on epidemiological characteristics, intervention feasibility, and economic cost. However, economic evaluations of NPIs are rarely being performed. A study in South Africa has suggested that a combination of interventions could be cost-effective (US$ 340 per lifetime years of lives saved). However, it is difficult to generalize the findings because of the different resources, threats, and living environments (5).

The suppression and mitigation were main non-pharmaceutical measures (6). This study aimed to assess the cost-effectiveness of different public health measures and provide suggestions and assist authorities and policymakers in making better decisions and resource allocations in the fight against the COVID-19 outbreak in China.

## Methods

### Model

A stochastic agent-based model (ABM) was used to simulate the COVID-19 outbreak with different interventions. NetLogo software (Wilensky, Northwestern University) was used to build the model and run the simulation. We constructed the domain with the total number of agents, following a susceptible-latent-infectious-recovered (SLIR) framework. In the domain, each agent was initially susceptible, and then COVID-19 cases were introduced into the agents. The infectious agent can infect susceptible agents with an infectious ability following the distance transmission probability function of β (*r*) (7). The simulation stopped when there was no exposed or infected agent in the space. We assumed that the recovered agents would not become susceptible again. One or four COVID-19 cases were introduced randomly to the simulated space with 2,000 individuals in two scenarios to represent sporadic (one imported case) and cluster (four imported cases) outbreaks.

### Comparator Strategies

In this study, we included measures for personal protection, isolation-and-quarantine, and community containment. These single measures were combined to develop various joint intervention strategies. Two joint interventions were formulated: (1) program A: personal protection and isolation-and-quarantine; (2) program B: personal protection and community containment. We compared different single and joint strategies vs. no interventions.

Systematic reviews have proved that it is useful to reduce the transmission of respiratory viruses through personal physical interventions (8–10). In our study, we defined personal protection as mask-wearing and frequent handwashing. Isolation was defined as the isolation of symptomatic and infected individuals and quarantine as the tracing and quarantine of close contacts of symptomatic and infected individuals for a certain period (11, 12). These practical tools have been used for hundreds of years in the fight against infectious diseases (13). In previous outbreaks, such as SARS in 2003 and Ebola in 2014, controlling the spread of infectious diseases has been proven effective by using isolation-and-quarantine (14–17).

Community containment is a form of social distancing, which was designed to reduce personal interaction and thereby transmission risk (18). In this study, the enforcement of community containment was a restriction on the movement of people within a community, thereby minimizing human contact (19).

### Epidemiological Parameter

The incubation period and serial interval came from the estimation of the Chinese Center for Disease Control and Prevention (China CDC) and Guangdong Provincial CDC in the fieldwork (20, 21) and were considered fitting for the gamma distribution in the model (3). The parameter of distance transmission probability has been reported in a previous study (7). The protective effectiveness of personal physical interventions was derived from cluster randomized controlled trials (22). In our study, we converted the odds ratio (OR) of handwashing and mask-wearing into the relative risk (RR) and calculated the (1–RR)/RR as personal protection effectiveness (23).

In the model, we set the probability and delay time for isolation-and-quarantine. The isolation delay time referred to the time dealing with patients lagged behind the infection onset; the quarantine delay time referred to the time of handling close contacts lagged behind the time of exposure. Initially, we assumed that the index case (initial imported case) would be 100% isolated with no time delay (infecting others and isolation were conducted within the same day, and infecting others preceded isolation). The quarantine probability was 100%, and the delay time was 2 days. In the sensitivity analysis, the probability of quarantine of close contacts was set between 25 and 100%, and the delay time was set between 0 and 21 days (3 weeks).

### Cost

The economic data were derived from fieldwork and previous literature (Table 1). The cost of personal protection included masks and handwashing (water and soap). The price of the mask was set at US$ 0.14, and we assumed that two masks were used per person per day (24). Given that soap would be used for handwashing, the cost of handwashing per person per day was calculated as the formula provided in the previous study (29):


$$\begin{array}{l} Cost_{pp}=f\times v\times C_{water}+\left(C_{soap}/t\right) \end{array}$$

**Table 1:** Parameters in the ABM model.

**Parameter**	**Base-value**	**Distribution**	**Sources**
**Model set**			
Initial agents	2,000	–	Assumption
Background transmission constant	1	–	(7)
Infect radius	1	–	(7)
Exponent in transmission rates	2	–	(7)
**Epidemiology**			
Serial interval (days)	Mean:7.5; SD:3.4	Gamma	(20)
Incubation period (days)	Mean:4.8; SD:2.6	Gamma	(21)
Odds ratio of personal intervention	0.33	–	(22)
**Cost (US$)**			
Surgical mask (per unit)	0.14	–	(24)
Soap (per unit)	2.85	–	Field work
Water cost per liter	0.00041	–	Field work
Direct medical cost per case	6,500	–	(25, 26)
Quarantine of each close contact per day	50	–	Field work
**Other parameters**			
Hospitalization time (days)	17	–	(25, 26)
Rest time (days)	7	–	Assumption
Quarantine time (days)	14	–	Field work
Per capital disposable income (US$)	4,401	–	(27)
Per capital GDP (US$)	9,595	–	(28)

*ABM, agent-based model; GDP, gross domestic product*.

where Cost_pp_ is the cost of handwashing, *f* is the times of handwashing per day, which we set to 6, *v* is the volume of handwashing per time, which we set to 1,000 cc/ml, *C*_water_ is the water cost per liter and was US$ 0.00041, *C*_soap_ is the cost of soap and was US$ 2.85, and *t* is the number of days soap was available, which was set to 60. We assumed that the day of personal protection was equal to the time from the day of the first case to the day the last case in the area recovered plus 14 days.

The cost of the cases included direct medical costs and indirect costs. Based on previous studies, the average medical cost of each COVID-19 patient was estimated to be US$ 6,500 (25, 26). Referring to the human capital approach in disease burden (30), we estimated the indirect cost of infected patients using per capita disposable income (PCDI)/365.25^*^ (hospitalization days and added rest days). The average hospital stay ranged from 14 to 27 days (25, 26), and we set 17 days as the baseline value. The average rest time was estimated as 7 days. We assumed that the cost of isolation would be included in hospitalization costs. The cost of quarantine of close contacts included both direct and indirect costs. The cost of quarantine (daily accommodation and surveillance) per day was US$ 50 for each close contact. Similar to the human capital approach in disease burden (30), the indirect cost of quarantine of close contacts was calculated as PCDI/365.25^*^ days of quarantine. For COVID-19, the quarantine time for close contacts was ~14 days in China.

There is considerably limited research on providing an estimation method for the cost of community containment. Referring to the human capital, we posed the following formulas to roughly estimate the cost of community containment during the current COVID-19 outbreak in China.


$$\begin{array}{l} Cost_{cc}=t*n*\frac{PCDI}{365.25}*w \end{array}$$

where the Cost_cc_ is the cost of community containment, *t* is the days of containment, *n* is the total number of individuals in the space, PCDI is the per capita disposable income, and *w* is the weight of calculation, which we set to 0.8. We assumed that the day of containment was equal to the period from the day the first case occurred to the last case recovered in the region plus 14 days. We used the PCDI in 2018 from the National Bureau of Statistical (27). In our study, all costs (RMB) were converted into US$ based on the 2018 currency conversion rate, namely 1 US$ is the 6.879 RMB (http://www.safe.gov.cn/safe/2018/0928/10272.html).

### Measurement of Cost-Effectiveness

The main health benefits of our study were avoidance of infection by taking preventative measures vs. no intervention. The incremental cost-effectiveness ratios (ICERs)—the main cost-effectiveness outcome—were calculated as the difference in the total costs between the intervention and non-intervention cohorts divided by the difference in total avoided infections. Positive ICERs showed the incremental costs required to avoid an infected person. We used “cost-saving” to replace with reporting negative ICERs values to avoid wrong interpretations of negative ICERs. The strategy was considered cost-effective if the ICERs were lower than three times per capita GDP. In 2018, China's per capita GDP was US$ 9,595 (28). We did not discount the cost because of the short period of the analysis. We performed 1,000 Monte Carlo simulations and reported the mean and SD of the results of the runs. Reporting on the methods and results conformed to the Consolidated Health Economic Evaluation Reporting Standards (Supplementary Table A.1) (31).

One- and two-way sensitivity analyses were performed to explore the impact of the parameters in the range to test the robustness of the findings, including the epidemiological characteristics, intervention implementation, and economic parameters.

## Results

### Effectiveness of Measures in Scenario I (One Case)

With the introduction of one case, each strategy would avoid the number of cases and be cost-effective compared with no intervention (Table 2). Isolation-and-quarantine was the most cost-effective intervention, as it prevented 1,696 cases and saved US$ 11,515,944. The most effective single protective strategy was community containment, which avoided one more case than isolation-and-quarantine at an additional US$ 549,186. In other words, program A could avert one more case compared to single isolation-and-quarantine.

**Table 2:** The cost-effectiveness of intervention measures in different scenario (US$1,000)^*^.

**Scenario**	**Type**	**Intervention strategy**	**Number of cases**	**Cost**			**ICERs**
				**Cost of cases**	**Cost of measures**	**Total cost**	
I		No intervention	1,698 ± 716.41	11,528.37 ± 4,863.86	0	11,528.37 ± 4,863.86	-
	Single	Personal protection	1,319 ± 950.02	8,952.90 ± 6,449.89	486.97 ± 260.28	9,439.86 ± 6,700.97	cost-saving
		Isolation-and-Quarantine	2 ± 1.08	10.46 ± 7.38	1.97 ± 1.55	12.43 ± 8.40	cost-saving
		Community containment	1 ± 0.70	9.64 ± 4.75	551.97 ± 52.19	561.61 ± 53.21	cost-saving
	Joint	Program A	1 ± 0.47	9.23 ± 4.58	170.07 ± 22.89	179.30 ± 25.04	cost-saving
		Program B	1 ± 0.48	8.55 ± 3.29	712.60 ± 60.02	721.15 ± 60.79	cost-saving
II		No intervention	1,998 ± 2.00	13,564.99 ± 13.56	0	13,564.99 ± 13.56	
	Single	Personal protection	1,998 ± 2.17	13,562.34 ± 14.75	501.24 ± 54.21	14,063.58 ± 51.59	1,278.438
		Isolation-and-Quarantine	8 ± 2.26	52.34 ± 15.33	6.21 ± 2.61	58.56 ± 16.58	cost-saving
		Community containment	7 ± 1.91	49.90 ± 12.96	608.70 ± 59.26	658.60 ± 65.00	cost-saving
	Joint	Program A	7 ± 2.07	49.15 ± 14.05	189.54 ± 13.62	238.70 ± 22.76	cost-saving
		Program B	7 ± 1.75	48.81 ± 11.89	795.44 ± 86.62	844.25 ± 90.98	cost-saving

*ICERs, incremental cost-effectiveness ratios: compared with no intervention.*

* *Program A: personal protection and isolation-and-quarantine; Program B: personal protection and community containment*.

### Effectiveness of Measures in Scenario II (Four Cases)

In scenario II (Table 2), personal protection was not cost-effective when compared with no intervention (ICERs > three times per capita GDP). Isolation-and-quarantine was still the most cost-effective strategy, preventing 1,990 cases and saving US$ 13,372,397. Compared with isolation-and-quarantine, community containment could avoid one more case with an additional US$ 600,044. Similarly, program A vs. single isolation-and-quarantine could avert one more case.

### One-Way Sensitivity Analysis

#### Transmission Constant

The number of cases depended on the transmission constant in scenario I (Supplementary Table A.2); it remained stable after changing the transmission constant in scenario II (Supplementary Table A.3). The cases increased as the transmission constant increased in scenario I. The basic reproduction number (R0) was 1.84 (95%CI: 1.81, 1.87) and 3.80 (95%CI: 3.53, 4.06), respectively, when the transmission constant was one in scenarios I and II (Supplementary Table A.4). When the transmission constant was changed from 0.25 to 2, isolation-and-quarantine was the most cost-effective single intervention, and program A was the most cost-effective joint intervention.

#### Initial Introduced Cases

**Figure 1:**
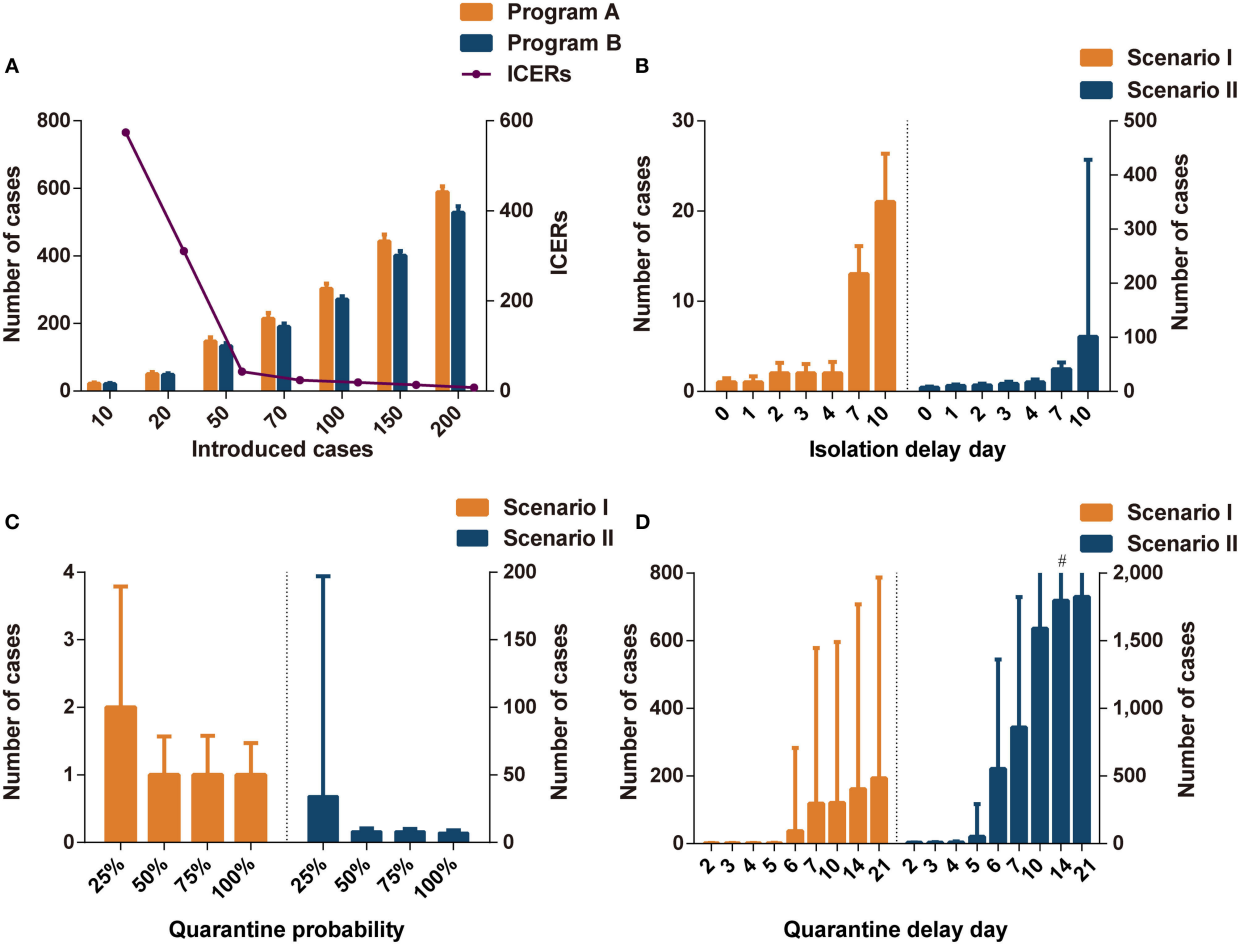
The impact of different parameters on interventions effectiveness. **(A)** The comparisons of infections in programs A and B in different introduced cases. **(B)** The impact of isolation delay day in program A in different scenarios. **(C)** The impact of quarantine probability in program A in different scenarios. **(D)** The impact of quarantine delay day in program A in different scenarios. Program A: personal protection and isolation-and-quarantine; Program B: personal protection and community containment. ICERs: incremental cost-effectiveness ratios (US$1,000 per case avoided). ^#^The interval extends out of the plotting region.

The number of imported cases was a key parameter influencing the effectiveness and cost-effectiveness of the analysis. There were no significant differences in effectiveness between programs A and B when the number of imported cases was set to 10 or 20 (Figure 1A and Supplementary Table A.5). When the number of imported cases was >50, program B (including community containment) could effectively decrease the cases compared to program A (including isolation-and-quarantine), but the former was not cost-effective. The threshold analysis showed that program B became cost-effective (ICERs < three times per capita GDP) compared to program A when initial cases increased to 65 imported cases (Supplementary Table A.6).

#### Isolation Delay Time

The isolation delay time did not contribute to the spread of infections in scenario I (Figure 1B); however, the increase in isolation delay time caused a significant increase in the number of infections in scenario II. When the isolation delay of the four index cases reached 4 days, more than 15 people were infected, which was three times higher than without isolation delay. When the isolation delay of the initial index cases reached 7 days in the scenario II, the program B was more cost-effective than program A; when the isolation delay reached 10 days in the scenario I, program B dominated program A (Supplementary Table A.7).

#### Quarantine Probability

The effectiveness of isolation-and-quarantine was sensitive to low quarantine probability. When the tracing probability of close contact was reduced to 25%, the number of infected people increased significantly, especially in scenario II (Figure 1C). In scenarios I and II, the effectiveness of outbreak control measures in programs A and B was similar when the probability of tracing was above 50% (Supplementary Tables A.8, A.9). In scenario I, program B was not cost-effective compared to program A. The ICERs of program B were close to three times per capita GDP when the quarantine probability was 25% in scenario II. The threshold analysis showed that program B became cost-effective (ICERs < three times per capita GDP) compared to program A when the quarantine probability was below 28% (Supplementary Table A.10).

#### Quarantine Delay Time

Varying the quarantine delay time from 0 to 4 days had little influence on averting infected cases (Figure 1D). When the tracing delay time of close contacts was extended to 6 days, the number of infected people increased significantly (Supplementary Tables A.11, A.12). In scenario II, when the quarantine delay time reached 6 days, more than 500 people were likely to be infected, accounting for a quarter of the space's population. Program B was more cost-effective than program A when the delay time was more than 5 days in scenario I and 4 days in scenario II (ICERs < three times per capita GDP).

#### Cost of Patients

Varying the cost of patients from US$ 2,900 to US$ 10,000, the ICERs of interventions compared to the non-intervention decreased (Supplementary Tables A.13, A.14). The most cost-effective strategy was isolation-and-quarantine in scenarios I and II.

### Two-Way Sensitivity Analysis

#### Transmission Constant and Quarantine Probability

In scenario I, the effectiveness of outbreak control was not sensitive to the transmission constant or quarantine probability (Supplementary Table A.15). When the transmission constant was set to two, the outbreak could be controlled by a 25% probability quarantine. However, as the transmission constant increased in scenario II, the control of the outbreak required a higher quarantine probability. When the quarantine probability was 25%, and the transmission constant was two, it was likely that about a quarter of the people would be infected in scenario II (Supplementary Table A.16). Program A was superior to program B in scenarios I and II. When the transmission constant was above one, and the quarantine probability was below 25%, program B was cost-effective (ICERs < three times per capita GDP).

#### Isolation Delay Time and Quarantine Probability

**Figure 2:**
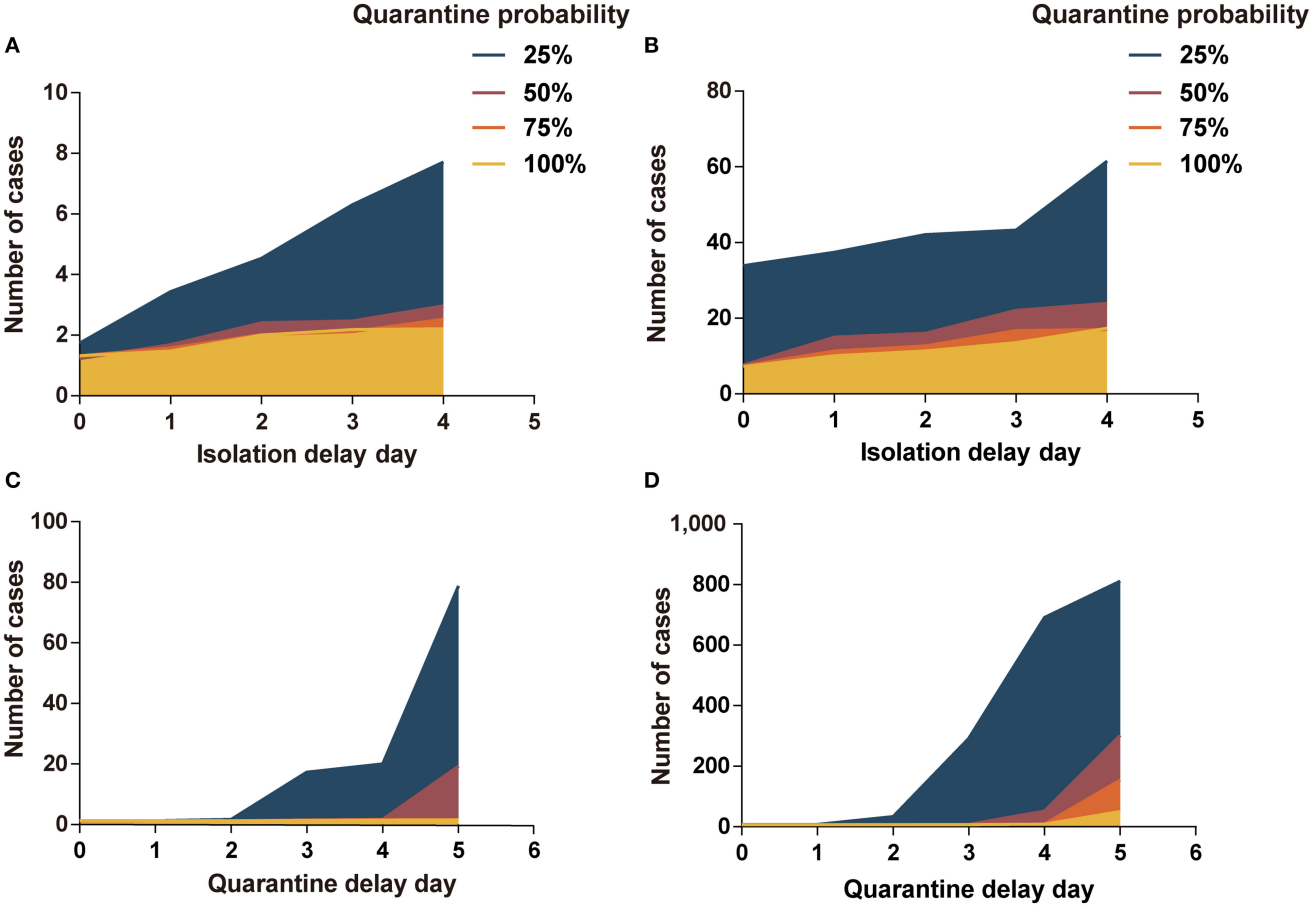
Impact of isolation-and-quarantine parameters on interventions effectiveness. **(A)** The impact of isolation delay time and quarantine probability in scenario I; **(B)** the impact of isolation delay time and quarantine probability in scenario II; **(C)** the impact of quarantine delay time and quarantine probability in scenario I; **(D)** the impact of quarantine delay time and quarantine probability in scenario II. Program A: personal protection and isolation-and-quarantine.

In scenario I, the quarantine probability and isolation delay time in the range of our analysis did not have a significant effect on the cost-effectiveness results. However, when the quarantine probability was 25% and the isolation delay time reached 3 or 4 days, the variability in the effect of infection control increased (Figure 2A and Supplementary Table A.17). In scenario II, the cases increased significantly with a decrease in quarantine probability and an increase in isolation delay time (Figure 2B and Supplementary Table A.18). In scenario II, when the quarantine probability decreased to 25%, program B was more cost-effective than program A (ICERs < three times per capita GDP). When the probability reached 50%, program B was cost-effective at an isolation delay time of more than 2 days.

#### Quarantine Delay Time and Quarantine Probability

The low quarantine probability and long quarantine delay time contributed to the outbreak of COVID-19, especially in scenario II (Figures 2C,D). At 25% probability and a 5-day delay time, there were more than 800 people infected in scenario II. The cases increased significantly as the quarantine probability decreased and the isolation delay time increased. In scenario I, program B was more cost-effective than program A when the probability reached 25% and the delay time was more than 2 days, or when the probability reached 50% and delay time was more than 4 days (Supplementary Table A.19). In scenario II, program B was cost-effective when the probability reached 25% or when the probability reached 50% and delay time was more than 3 days (Supplementary Table A.20).

#### Cost Parameter and Quarantine Probability

With an increase in the cost of quarantine per close contact per day, the ICERs of program B compared to those of program A decreased. In scenario I, program A was superior to program B (Supplementary Table A.21). In scenario II, program B was more cost-effective at 25% quarantine probability. Compared with program A, the ICERs of program B increased with an increase in the cost of community containment (Supplementary Table A.22). However, the optimal strategy was not affected by the cost of community containment.

#### Cost Parameter and Quarantine Delay Time

Similarly, the cost of quarantine and that of community containment were considered in the analyses (Supplementary Tables A.23, A.24). A change in the cost parameter did not affect the choice of the optimal strategy.

## Discussion

Our study provided an assessment of different measures to control the community transmission of COVID-19. In sporadic (one imported case) and cluster (four imported cases) outbreaks, the isolation of infectious cases and quarantine of individuals exposed to the infected were the most cost-effective measures. In the virtual environment, isolation-and-quarantine could significantly reduce the number of infections and avoid the disease outbreak at a lower cost. From the perspective of effectiveness and cost-effectiveness of controlling the spread of COVID-19, the joint strategy of personal protection and isolation-and-quarantine was the optimal choice, averting more cases than only isolation-and-quarantine.

In sporadic outbreaks, the effectiveness of isolation-and-quarantine was most sensitive to the quarantine delay time. A one-way analysis revealed a marked increase in the number of infections when the quarantine delay time reached 6 days. There was no significant difference in numbers in the sporadic area when the quarantine probability changed from 25 to 100%. However, the two-way analysis suggested that at 25% probability, more infections were likely to occur when the quarantine delay time was >2 days. In the cluster area, these parameters played an important role in the effectiveness of the interventions. The probability of contact tracing decreased, and the delay time of isolation-and-quarantine increased, leading to fewer cases averted by the intervention. The long delay time and low quarantine probability could accelerate the outbreak of COVID-19.

The effectiveness and cost-effectiveness of the interventions were sensitive to initial imported cases. The increase in imported cases could lead to an increase in the risk of COVID-19 infection, even when conducting strict interventions. We found that the cases avoided by isolation-and-quarantine and community containment were not significant when the imported cases were below 20. When the number of imported cases reached 50, community containment could avoid more cases significantly. A strategy that included community containment was cost-effective when imported cases reached 65, resulting in 3.25% of the community population (2,000 people) infected. This study found that the initial number of cases had an effect on the effectiveness of interventions (32).

The choice of optimal strategy depended on the setting of the intervention parameters. We compared the strategy of personal protection and isolation-and-quarantine (program A) with that of personal protection and community containment (program B). Generally, program A was more cost-effective than program B. However, program B was cost-effective at 25% probability and more than 2 quarantine delay days or 50% probability and >5 quarantine delay days in a sporadic outbreak area. Program B was better than program A at 25% quarantine probability or a quarantine delay time of more than 3 days in a cluster outbreak.

The effectiveness of isolation and contact tracing was associated with the extent of transmission before symptom onset (33). The proportion of asymptomatic infections contributes to the outbreak of COVID-19 (32), which is consistent with our findings. In our study, community containment would be more efficient and cost-effective when the quarantine delay time is greater than the latent period. We found that the increase in quarantine time delay was similar to the presence of asymptomatic infection. For asymptomatic infection or latent infection, failure to detect in time leads to the absence of isolation and continuation of transmission. We found that this phenomenon was similar to the low probability of quarantine or quarantine delay time, which caused the infection to continue to spread. The proportion of asymptomatic infections had a significant effect on the choice of controlling strategy.

There are some limitations to this study. First, the cost of societal interventions is difficult to estimate. In our study, a human capital approach was used, which might estimate the cost more conservatively. The cost of the disease could also be higher than the actual situation in China. Second, the generalizability of the model might be limited due to small simulated population. Third, more strategies need to be taken into consideration in the future, including “hybrid” type including different measures. For example, isolation-and-quarantine was used firstly and community containment would be taken when infected cases reached a threshold. Finally, the simplification of the model will have some biases compared with the real situation because the flow of people will be affected by many factors.

## Conclusions

Isolation-and-quarantine was the most cost-effective intervention in sporadic and cluster outbreaks of COVID-19. Personal protection, isolation, and quarantine were the optimal joint strategies to prevent more cases than single isolation-and-quarantine strategies. Rapid and effective isolation-and-quarantine can control the outbreak of COVID-19. The community containment could be more cost-effective as the efficiency of isolation-and-quarantine decreases and the outbreak increases in size.

## Data Availability Statement

The original contributions presented in the study are included in the article/Supplementary Material, further inquiries can be directed to the corresponding author.

## Author Contributions

HuJ and QW conceived and designed the study. HuJ, QW, NS, JH, TC, and LY designed the model. QW, JA, HoJ, KX, and CB collected the parameters. QW, NS, and JH did the data analyses. QW, HuJ, NS, and TA contributed to the writing of the manuscript. All authors interpreted the results and approved the final version for publication.

## Funding

This work was supported by the Jiangsu Provincial Major Science and Technology Demonstration Project (BE2017749); Southeast University Novel coronavirus research (3225002001C1); Postgraduate Research and Practice Innovation Program of Jiangsu Province (KYCX20_0153) and Jiangsu Provincial Six Talent Peak (WSN-002). The funder of the study had no role in the design of the study and collection, analysis, and interpretation of data and in writing the manuscript.

## Conflict of Interest

The authors declare that the research was conducted in the absence of any commercial or financial relationships that could be construed as a potential conflict of interest.

## Publisher's Note

All claims expressed in this article are solely those of the authors and do not necessarily represent those of their affiliated organizations, or those of the publisher, the editors and the reviewers. Any product that may be evaluated in this article, or claim that may be made by its manufacturer, is not guaranteed or endorsed by the publisher.

## References

[1] World Health Organization. Coronavirus disease 2019. (COVID-19) Situation Report. November 23, (2021). Available online at: https://www.who.int/publications/m/item/weekly-operational-update-on-covid-19-−23-november-2021 (accessed November 23, 2021).

[2] Kwok KO Tang A Wei VWI Park WH Yeoh EK Riley S. Epidemic e. Comput Struct Biotechnol J. (2019) 17:186–94. 10.1016/j.csbj.2019.01.003 30809323 PMC6376160

[3] Broughel J Kotrous M. The benefits of coronavirus suppression: A cost-benefit analysis of the response to the first wave of COVID-19 in the United States. PLoS ONE. (2021) 16:e0252729. 10.1371/journal.pone.0252729 34081757 PMC8174714

[4] Gopinath G. The great lockdown: worst economic downturn since the Great Depression. International Monetary Fund. Available online at: https://blogs.imf.org/2020/04/14/ the-great-lockdown-worst-economic-downturn-since-the-great-depression/ (accessed April 14, 2020).

[5] Kellerborg K Brouwer W van Baal P. Costs and benefits of interventions aimed at major infectious disease threats: lessons from the literature. Eur J Health Econ. (2020) 21:1329–50. 10.1007/s10198-020-01218-4 32789780 PMC7425274

[6] Neil M Ferguson. Report 9 - Impact of non-pharmaceutical interventions (NPIs) to reduce COVID-19 mortality and healthcare demand. (2020). Available online at: https://www.imperial.ac.uk/mrc-global-infectious-disease-analysis/covid-19/report-9-impact-of-npis-on-covid-19/ (accessed November 23, 2021). 10.1007/s11538-020-00726-x PMC7140590 32270376

[7] Kim Y Ryu H Lee S. Agent-based modeling for super-spreading events: a case study of MERS-CoV transmission dynamics in the Republic of Korea. Int J Environ Res Public Health. (2018) 15:2369. 10.3390/ijerph15112369 30373151 PMC6265857

[8] Jefferson T Del Mar CB Dooley L Ferroni E Al-Ansary LA Bawazeer GA. Physical interventions to interrupt or reduce the spread of respiratory viruses. Cochrane Database Syst Rev. (2011) 2011:CD006207. 10.1002/14651858.CD006207.pub4 21735402 PMC6993921

[9] Saunders-Hastings P Crispo JAG Sikora L Krewski D. Effectiveness of personal protective measures in reducing pandemic influenza transmission: A systematic review and meta-analysis. Epidemics. (2017) 20:1–20. 10.1016/j.epidem.2017.04.003 28487207

[10] Xiao J Shiu EYC Gao H Wong JY Fong MW Ryu S. Nonpharmaceutical measures for pandemic influenza in nonhealthcare settings-personal protective and environmental measures. Emerging Infect Dis. (2020) 26:10.3201/eid2605.190994. 10.3201/eid2605.190994 32027586 PMC7181938

[11] Peak CM Childs LM Grad YH Buckee CO. Comparing nonpharmaceutical interventions for containing emerging epidemics. Proc Natl Acad Sci USA. (2017) 114:4023–8. 10.1073/pnas.1616438114 28351976 PMC5393248

[12] Mitka M. SARS thrusts quarantine into the limelight. JAMA. (2003) 290:1696–8. 10.1001/jama.290.13.1696 14519692

[13] Svoboda T Henry B Shulman L Kennedy E Rea E Ng W. Public health measures to control the spread of the severe acute respiratory syndrome during the outbreak in Toronto. N Engl J Med. (2004) 350:2352–61. 10.1056/NEJMoa032111 15175437

[14] Eames KTD Keeling MJ. Contact tracing and disease control. Proc Biol Sci. (2003) 270:2565–71. 10.1098/rspb.2003.2554 14728778 PMC1691540

[15] Grigg C Waziri NE Olayinka AT Vertefeuille JF Centers for disease C. prevention Use of group quarantine in ebola control - Nigeria, 2014. MMWR Morb Mortal Wkly Rep. (2015) 64:124. 25674994 PMC4584688

[16] Centers for Disease Control and Prevention. Use of quarantine to prevent transmission of severe acute respiratory syndrome–Taiwan, 2003. MMWR Morb Mortal Wkly Rep. (2003) 52:680−3. 12881699

[17] Centers for Disease Control and Prevention. Efficiency of quarantine during an epidemic of severe acute respiratory syndrome–Beijing, China, 2003. MMWR Morb Mortal Wkly Rep. (2003) 52:1037–40. 14586295

[18] Maharaj S Kleczkowski A. Controlling epidemic spread by social distancing: Do it well or not at all. BMC Public Health. (2012) 12. 10.1186/1471-2458-12-679 22905965 PMC3563464

[19] Centers for Disease Control and Prevention. Interventions for Community Containment. (2005). Available online at: https://www.cdc.gov/sars/guidance/d-quarantine/app1.html (accessed Feb 24, 2020).

[20] Li Q Guan X Wu P Wang X Zhou L Tong Y. Early transmission dynamics in Wuhan, China, of novel coronavirus-infected pneumonia. N Engl J Med. 382:1199–207. 10.1056/NEJMoa2001316 31995857 PMC7121484

[21] Liu T Hu J Kang M Lin L Zhong H Xiao J. Transmission dynamics of 2019 novel coronavirus (2019-nCoV). BioRxiv. (2020). 10.1101/2020.01.25.919787

[22] Cowling BJ Chan K-H Fang VJ Chen CKY Fung ROP Wai W. Facemasks and hand hygiene to prevent influenza transmission in households: a cluster randomized trial. Ann Intern Med. (2009) 151:437–46. 10.7326/0003-4819-151-7-200910060-00142. 10.7326/0003-4819-151-7-200910060-00142 19652172

[23] Zhang J Yu KF. What's the relative risk? A method of correcting the odds ratio in cohort studies of common outcomes. JAMA. (1998) 280:1690-1. 10.1001/jama.280.19.1690 9832001

[24] Mukerji S MacIntyre CR Seale H Wang Q Yang P Wang X. Cost-effectiveness analysis of N95 respirators and medical masks to protect healthcare workers in China from respiratory infections. BMC Infect Dis. (2017) 17:464. 10.1186/s12879-017-2564-9 28673259 PMC5496227

[25] Tang ZQ Li YT Yan H Ren TL Chen MJ. Comparative analysis of hospitalization expenses of 118 cases of pneumonia infected by New Coronavirus. J Nanjing Med Univ. (2021) 21:171–5.

[26] Wu D Chen Q Gu FJ Zhang DW Hu XS Qin FQ Mo GQ. Influencing factors of medical expenses for COVID-19 in patients. Acad J Chin PLA Med Sch. (2020) 41:1167–71.34040465

[27] National Bureau of the Statistics. Stable growth in household income and consumption. (2020). Available online at: http://www.stats.gov.cn/tjsj/zxfb/202001/t20200119_1723769.html (accessed Febuary 13, 2020).

[28] National Bureau of the Statistics. National Data. (2019). Available online at: http://data.stats.gov.cn/search.htm?s=GDP (accessed Febuary 27, 2020).

[29] Chen SC Liao CM. Cost-effectiveness of influenza control measures: a dynamic transmission model-based analysis. Epidemiol Infect. (2013) 141:2581–94. 10.1017/S0950268813000423 23481024 PMC9151383

[30] Zhou F Shefer A Wenger J Messonnier M Wang LY Lopez A. Economic evaluation of the routine childhood immunization program in the United States, 2009. Pediatrics. (2014) 133:577–85. 10.1542/peds.2013-0698 24590750

[31] Husereau D Drummond M Petrou S Carswell C Moher D Greenberg D. Consolidated health economic evaluation reporting standards (CHEERS) statement. BMJ (Clinical research ed). (2013) 346:f1049-f. 10.1136/bmj.f1049 23529982

[32] Hellewell J Abbott S Gimma A Bosse NI Jarvis CI Russell TW. Feasibility of controlling COVID-19 outbreaks by isolation of cases and contacts. Lancet Glob Health. (2020) 8:e488–96. 10.1016/S2214-109X(20)30074-7 32119825 PMC7097845

[33] Wilder-Smith A Chiew CJ Lee VJ. Can we contain the COVID-19 outbreak with the same measures as for SARS? Lancet Infect Dis. (2020) 20:e102–07. 10.1016/S1473-3099(20)30129-8 32145768 PMC7102636

