# Supplementary material for: Cost-Effectiveness of Public Health Measures to Control COVID-19 in China: A Microsimulation Modeling Study
Source: Front Public Health. 2022 Jan 4;9:726690. doi: 10.3389/fpubh.2021.726690 (PMC8763804; doi:10.3389/fpubh.2021.726690)
Supplement: Supplementary file 1 [file Table_1.DOCX]

**Additional file**

**Table A.1.** CHEERS checklist-Items. **Table A.2** The impact of transmission constant in scenario I (US$1,000). **Table A.3** The impact of transmission constant in scenario II (US$1,000). **Table A.4** Different Re of transmission constant in various scenarios. **Table A.5** Comparisons of impact of initial introduced cases (US$1,000). **Table A.6** Threshold analysis of cost-effectiveness of initial introduced cases (US$1,000). **Table A.7** The impact of isolation delay time in different scenarios (US$1,000). **Table A.8** The impact of quarantine probability in scenario I (US$1,000). **Table A.9** The impact of quarantine probability in scenario II (US$1,000). **Table A.10** Threshold analysis of cost-effectiveness of quarantine probability (US$1,000). **Table A.11** Comparisons of impact of various delay time in scenario I (US$1,000). **Table A.12** Comparisons of impact of various delay time in scenario II (US$1,000)**. Table A.13** Comparisons of impact of cost of patient in scenario I (US$1,000). **Table A.14** Comparisons of impact of cost of patient in scenario II (US$1,000)**. Table A.15** The impact of transmission constant and quarantine probability in scenario I (US$1,000). **Table A.16** The impact of transmission constant and quarantine probability in scenario II (US$1,000). **Table A.17** The impact of isolation delay time and quarantine probability in scenario I (US$1,000). **Table A.18** The impact of isolation delay time and quarantine probability in scenario II (US$1,000). **Table A.19** The impact of quarantine delay time and probability in scenario I (US$1,000). **Table A.20** The impact of quarantine delay time and probability in scenario II (US$1,000). **Table A.21** The impact of cost of quarantine and quarantine probability (US$1,000). **Table A.22** The impact of cost of community containment and quarantine probability (US$1,000). **Table A.23** The impact of cost of quarantine and quarantine delay time (US$1,000). **Table A.24** The impact of cost of community containment and quarantine delay time (US$1,000).

**Table A.1 CHEERS checklist-Items**

| **Section** | **Item No** | **Recommendation** | **Reported on page No/line No** |
| --- | --- | --- | --- |
| **Title and Abstract** |  |  |  |
| Title | 1 | Identify the study as an economic evaluation or use more specific terms such as “cost-effectiveness analysis”, and describe the interventions compared. | 1 |
| Abstract | 2 | Provide a structured summary of objectives, perspective, setting, methods (including study design and inputs), results (including base case and uncertainty analyses), and conclusions. | 1-2 |
| **Introduction** |  |  |  |
| Background and objectives | 3 | Provide an explicit statement of the broader context for the study.  Present the study question and its relevance for health policy or practice decisions. | 3 |
| **Methods** |  |  |  |
| Target population and subgroups | 4 | Describe characteristics of the base case population and subgroups analysed, including why they were chosen. | - |
| Setting and location | 5 | State relevant aspects of the system(s) in which the decision (s) need(s) to be made. | 4 |
| Study perspective | 6 | Describe the perspective of the study and relate this to the costs being evaluated. | - |
| Comparators | 7 | Describe the interventions or strategies being compared and state why they were chosen. | 5 |
| Time horizon | 8 | State the time horizon(s) over which costs and consequences are being evaluated and say why appropriate. | - |
| Discount rate | 9 | Report the choice of discount rate(s) used for costs and outcomes and say why appropriate. | 9 |
| Choice of health outcomes | 10 | Describe what outcomes were used as the measure(s) of benefit in the evaluation and their relevance for the type of analysis performed. | 9 |
| Measurement of effectiveness | 11a | *Single study-based estimates*: Describe fully the design features of the single effectiveness study and why the single study was a sufficient source of clinical effectiveness data. | 9 |
|  | 11b | *Synthesis-based estimates*: Describe fully the methods used for identification of included studies and synthesis of clinical  effectiveness data. | - |
| Measurement and valuation of preference  based outcomes | 12 | If applicable, describe the population and methods used to elicit preferences for outcomes. | - |
| Estimating resources  and costs | 13a | *Single study-based economic evaluation*: Describe approaches used to estimate resource use associated with the alternative interventions. Describe primary or secondary research methods for valuing each resource item in terms of its unit cost. Describe any adjustments made to approximate to opportunity costs. | - |
|  | 13b | *Model-based economic evaluation*: Describe approaches and data sources used to estimate resource use associated with model health states. Describe primary or secondary research methods for valuing each resource item in terms of its unit cost. Describe any adjustments made to approximate to opportunity costs. | 10 |
| Currency, price date,  and conversion | 14 | Report the dates of the estimated resource quantities and unit costs. Describe methods for adjusting estimated unit costs to the year of reported costs if necessary. Describe methods for converting costs into a common currency base and the exchange  rate. | 7-8 |
| Choice of model | 15 | Describe and give reasons for the specific type of decision analytical model used. Providing a figure to show model structure is strongly recommended. | - |
| Assumptions | 16 | Describe all structural or other assumptions underpinning the decision-analytical model. | 4-5 |
| Analytical methods | 17 | Describe all analytical methods supporting the evaluation. This could include methods for dealing with skewed, missing, or censored data; extrapolation methods; methods for pooling data; approaches to validate or make adjustments (such as half cycle corrections) to a model; and methods for handling population heterogeneity and uncertainty. | 9 |
| **Results** |  |  |  |
| Study parameters | 18 | Report the values, ranges, references, and, if used, probability distributions for all parameters. Report reasons or sources  for distributions used to represent uncertainty where appropriate. Providing a table to show the input values is strongly recommended. | 7-9 |
| Incremental costs and  outcomes | 19 | For each intervention, report mean values for the main categories of estimated costs and outcomes of interest, as well as mean differences between the comparator groups. If applicable, report incremental cost-effectiveness ratios. | 10 |
| Characterizing uncertainty | 20a | *Single study-based economic evaluation*: Describe the effects of sampling uncertainty for the estimated incremental cost and incremental effectiveness parameters, together with the impact of methodological assumptions (such as discount rate, study perspective). | - |
|  | 20b | *Model-based economic evaluation*: Describe the effects on the results of uncertainty for all input parameters, and uncertainty related to the structure of the model and assumptions. | 11-15 |
| Characterizing heterogeneity | 21 | If applicable, report differences in costs, outcomes, or cost-effectiveness that can be explained by variations between subgroups of patients with different baseline characteristics or other observed variability in effects that are not reducible by more information. | 11-15 |
| **Discussion** |  |  |  |
| Study findings, limitations,  generalizability,  and current  knowledge | 22 | Summarise key study findings and describe how they support  the conclusions reached. Discuss limitations and the generalisability of the findings and how the findings fit with current knowledge. | 15-17 |
| **Other** |  |  |  |
| Source of funding | 23 | Describe how the study was funded and the role of the funder in the identification, design, conduct, and reporting of the analysis. Describe other non-monetary sources of support. | 20 |
| Conflicts of interest | 24 | Describe any potential for conflict of interest of study contributors in accordance with journal policy. In the absence of a journal policy, we recommend authors comply with International Committee of Medical Journal Editors recommendations. | 20 |

“-”: not applicable

**Table A.2 The impact of transmission constant in scenario I (US$1,000)**

| Strategy |  | Transmission constant | | | | |
| --- | --- | --- | --- | --- | --- | --- |
|  |  | 0.25 | 0.50 | 0.75 | 1 | 2 |
| No intervention | Number of cases | 401±801.43 | 1,258±968.17 | 1,630±776.59 | 1,698±716.41 | 1,978±939.32 |
|  | Cost of cases | 2,720.97±5441.06 | 8,543.03±6,573.08 | 11,659.81±4,724.81 | 11,067.75±5,272.42 | 13,425.81±1,355.70 |
|  | Cost of measures | 0 | 0 | 0 | 0 | 0 |
|  | Total cost | 2,720.97±5441.06 | 8,543.03±6,573.08 | 11,659.81±4,724.81 | 11,067.75±5,272.42 | 13,425.81±1,355.70 |
| Isolation-and-quarantine | Number of cases | 1±0.38 | 1±0.45 | 1±0.57 | 2±1.08 | 2±0.94 |
|  | Cost of cases | 7.74±2.56 | 8.42±3.07 | 9.10±3.89 | 10.46±7.38 | 12.42±6.40 |
|  | Cost of measures | 2.32±1.34 | 1.96±1.35 | 2.05±1.57 | 1.97±1.55 | 1.60±1.34 |
|  | Total cost | 10.06±3.06 | 10.38±3.68 | 11.15±4.32 | 12.43±8.40 | 14.02±6.96 |
|  | ICERs | cost-saving | cost-saving | cost-saving | cost-saving | cost-saving |
| Program A | Number of cases | 1±0.22 | 1±0.37 | 1±0.45 | 1±0.47 | 1±0.69 |
|  | Cost of cases | 7.13±1.49 | 7.67±2.49 | 7.94±3.06 | 9.23±4.58 | 9.91±4.67 |
|  | Cost of measures | 165.67±23.63 | 169.39±18.72 | 167.12±22.20 | 170.07±22.89 | 168.56±22.92 |
|  | Total cost | 172.80±23.82 | 177.07±19.22 | 175.06±22.86 | 179.30±25.04 | 178.47±25.00 |
|  | ICERs | cost-saving | cost-saving | cost-saving | cost-saving | cost-saving |
| Program B | Number of cases | 1±0.37 | 1±0.40 | 1±0.37 | 1±0.48 | 1±0.73 |
|  | Cost of cases | 7.67±2.49 | 7.94±2.74 | 7.67±2.49 | 8.55±3.29 | 10.05±4.97 |
|  | Cost of measures | 712.32±50.29 | 711.79±64.33 | 699.43±67.24 | 712.60±60.02 | 719.64±55.54 |
|  | Total cost | 720.00±50.77 | 719.73±64.85 | 707.10±67.64 | 721.15±60.79 | 729.69±57.10 |
|  | ICERs | cost-saving | cost-saving | cost-saving | cost-saving | cost-saving |

Program A: personal protection and isolation-and-quarantine; Program B: personal protection and community containment; ICERs (incremental cost-effectiveness ratios): compared to no intervention.

**Table A.3 The impact of transmission constant in scenario II (US$1,000)**

| Strategy |  | Transmission constant | | | | |
| --- | --- | --- | --- | --- | --- | --- |
|  |  | 0.25 | 0.50 | 0.75 | 1 | 2 |
| No intervention | Number of cases | 1,998±1.48 | 1,998±2.00 | 1,998±1.91 | 1,998±2.00 | 1,998±1.96 |
|  | Cost of cases | 13,565.47±10.69 | 13,562.75±13.60 | 13,566.15±12.96 | 13,564.99±13.56 | 13,567.84±13.30 |
|  | Cost of measures | 0 | 0 | 0 | ±0 | 0 |
|  | Total cost | 13,565.47±10.69 | 13,562.75±13.60 | 13,566.15±12.96 | 13,564.99±13.56 | 13,567.84±13.30 |
| Isolation-and-quarantine | Number of cases | 5±1.12 | 6±1.60 | 7±1.89 | 8±2.26 | 9±2.71 |
|  | Cost of cases | 35.64±7.62 | 43.11±10.85 | 48.61±12.83 | 52.34±15.33 | 61.31±18.37 |
|  | Cost of measures | 7.23±2.77 | 7.03±2.67 | 6.31±2.43 | 6.21±2.61 | 5.86±2.58 |
|  | Total cost | 42.87±8.34 | 50.14±12.05 | 54.92±13.83 | 58.56±16.58 | 67.16±19.75 |
|  | ICERs | cost-saving | cost-saving | cost-saving | cost-saving | cost-saving |
| Program A | Number of cases | 5±0.95 | 6±1.52 | 6±1.60 | 7±2.07 | 9±2.29 |
|  | Cost of cases | 33.88±6.44 | 39.65±10.29 | 42.50±10.86 | 49.15±14.05 | 58.25±15.57 |
|  | Cost of measures | 185.58±13.06 | 188.37±14.29 | 189.87±18.93 | 189.54±13.62 | 194.55±15.25 |
|  | Total cost | 219.45±16.61 | 228.02±21.18 | 232.37±25.70 | 238.70±22.76 | 249.80±24.74 |
|  | ICERs | cost-saving | cost-saving | cost-saving | cost-saving | cost-saving |
| Program B | Number of cases | 5±0.95 | 6±1.33 | 7±1.72 | 7±1.75 | 8±2.12 |
|  | Cost of cases | 33.54±6.46 | 39.78±9.05 | 44.27±11.68 | 48.81±11.89 | 56.42±14.37 |
|  | Cost of measures | 760.97±61.75 | 780.91±88.43 | 785.87±76.39 | 795.44±86.62 | 804.46±76.96 |
|  | Total cost | 794.51±64.26 | 820.69±91.26 | 830.14±80.70 | 844.25±90.98 | 860.97±81.92 |
|  | ICERs | cost-saving | cost-saving | cost-saving | cost-saving | cost-saving |

Program A: personal protection and isolation-and-quarantine; Program B: personal protection and community containment; ICERs (incremental cost-effectiveness ratios): compared to no intervention.

**Table A.4 Different Re of transmission constant in various scenarios**

| Scenario |  | Transmission constant | | | | |
| --- | --- | --- | --- | --- | --- | --- |
|  |  | 0.25 | 0.50 | 0.75 | 1 | 2 |
| I | R0 (95%CI) | 1.29  (1.25, 1.33) | 1.65  (1.62, 1.68) | 1.77  (1.74, 1.80) | 1.84  （1.81, 1.87） | 2.08  (2.03, 2.16) |
| II | R0 (95%CI) | 3.08  (2.86, 3.30) | 3.18  (2.96, 3.39) | 3.51  (3.27, 3.74) | 3.80  (3.53, 4.06) | 3.83  (3.56, 4.11) |

R0: basic reproduction number

.

**Table A.5 Comparisons of impact of initial introduced cases (US$1,000)**

| Strategy |  | Imported cases | | | |  |  |  |
| --- | --- | --- | --- | --- | --- | --- | --- | --- |
|  |  | 10 cases | 20 cases | 50 cases | 70 cases | 100 cases | 150 cases | 200 cases |
| Program A | Number of cases | 22±3.78 | 50±6.54 | 147±12.29 | 214±17.53 | 303±15.08 | 443±20.56 | 589±17.17 |
|  | Cost of cases | 149.09±25.71 | 340.68±44.41 | 1,000.79±93.42 | 1,455.87±119.02 | 2,054.07±102.41 | 3,008.97±139.58 | 3,998.15±116.55 |
|  | Cost of measures | 213.44±18.11 | 240.82±19.53 | 278.51±19.00 | 198.61±23.68 | 395.98±29.44 | 443.64±22.23 | 479.25±18.44 |
|  | Total cost | 362.53±37.14 | 581.50±54.31 | 1,279.30±88.08 | 1,754.48±132.12 | 2,450.04±114.71 | 3,452.61±149.04 | 4,477.40±114.80 |
| Program B | Number of cases | 21±3.50 | 48±5.16 | 133±9.02 | 190±10.54 | 271±10.24 | 401±13.99 | 528±19.24 |
|  | Cost of cases | 141.62±23.77 | 325.95±35.04 | 889.77±61.23 | 1,291.98±71.53 | 1,838.85±69.49 | 2,723.14±94.99 | 3,581.29±130.66 |
|  | Cost of measures | 852.53±88.62 | 929.60±90.55 | 1,023.13±76.05 | 1,048.78±70.62 | 1,231.10±85.03 | 1,303.78±65.46 | 1,374.32±80.79 |
|  | Total cost | 994.15±92.10 | 1,255.55±102.58 | 1,922.90±105.46 | 2,340.76±101.92 | 3,069.95±119.94 | 4,026.92±93.17 | 4,955.61±163.88 |
|  | ICERs | 574.200 | 310.623 | 43.252 | 24.287 | 19.372 | 13.674 | 7.840 |

* Program A: personal protection and isolation-and-quarantine; Program B: personal protection and community containment; ICERs (incremental cost-effectiveness ratios): compared to Program A.

**Table A.6 Threshold analysis of cost-effectiveness of initial introduced cases (US$1,000)**

| Strategy |  | 60 cases | 62 cases | 63cases | 64cases | 65cases |
| --- | --- | --- | --- | --- | --- | --- |
| Program A | Number of cases | 179±15.22 | 182±13.53 | 189±15.17 | 191±15.39 | 196±16.72 |
|  | Total cost | 1,501.15±113.07 | 1,522.28±100.49 | 1,571.85±111.11 | 1,591.27±112.17 | 1,628.68±123.45 |
| Program B | Number of cases | 160.94±10.51 | 166±9.00 | 170±9.32 | 171±11.38 | 174±9.52 |
|  | Total cost | 2,116.01±1,129.94 | 2,170.30±106.17 | 2,194.72±94.63 | 2,200.77±108.04 | 2,220.59±68.14 |
|  | ICERs | 34.758 | 42.134 | 33.796 | 30.233 | 27.413 |

* ICERs (incremental cost-effectiveness ratios): compared with Program A.

**Table A.7 The impact of isolation delay time in different scenarios (US$1,000)**

| Scenario | Strategy | Isolation delay day | Number of cases | Cost | | | ICERs |
| --- | --- | --- | --- | --- | --- | --- | --- |
|  |  |  |  | Cost of cases | Cost of measures | Total cost |  |
| I | Program A | 0 | 1±0.47 | 8.35±3.18 | 169.08±25.67 | 177.43±26.86 | dominating |
|  | Program B |  | 1±0.48 | 8.55±3.29 | 712.60±60.02 | 721.15±60.79 | - |
|  | Program A | 1 | 1±0.67 | 10.05±4.56 | 181.08±19.11 | 191.13±20.72 | - |
|  | Program B |  | 1±0.48 | 8.55±3.29 | 712.60±60.02 | 721.15±60.79 | 2,409.204 |
|  | Program A | 2 | 2±1.17 | 13.58±7.94 | 187.50±16.97 | 201.08±21.31 | - |
|  | Program B |  | 1±0.48 | 8.55±3.29 | 712.60±60.02 | 721.15±60.79 | 702.805 |
|  | Program A | 3 | 2±1.03 | 14.87±13.79 | 194.84±13.79 | 209.70±17.30 | - |
|  | Program B |  | 1±0.48 | 8.55±3.29 | 712.60±60.02 | 721.15±60.79 | 549.946 |
|  | Program A | 4 | 2±1.27 | 15.00±8.63 | 195.19±18.85 | 210.20±23.39 | - |
|  | Program B |  | 1±0.48 | 8.55±3.29 | 712.60±60.02 | 721.15±60.79 | 537.850 |
|  | Program A | 7 | 13±3.11 | 91.44±20.30 | 223.56±13.95 | 315.00±34.66 |  |
|  | Program B |  | 1±0.48 | 8.55±3.29 | 712.60±60.02 | 721.15±60.79 | 33.85 |
|  | Program A | 10 | 21±5.35 | 144.65±34.75 | 843.53±86.62 | 888.19±94.53 |  |
|  | Program B |  | 1±0.48 | 8.55±3.29 | 712.60±60.02 | 721.15±60.79 | cost-saving |
| II | Program A | 0 | 7±2.07 | 49.15±14.05 | 189.54±13.62 | 238.70±22.76 | - |
|  | Program B |  | 7±1.87 | 48.81±11.89 | 795.44±86.62 | 844.25±90.98 | 12,111.151 |
|  | Program A | 1 | 10±2.86 | 68.57±19.44 | 204.29±16.88 | 272.86±29.31 | - |
|  | Program B |  | 7±1.87 | 48.81±11.89 | 795.44±86.62 | 844.25±90.98 | 196.356 |
|  | Program A | 2 | 11±3.38 | 77.06±22.92 | 211.91±13.86 | 288.97±31.98 | - |
|  | Program B |  | 7±1.87 | 48.81±11.89 | 795.44±86.62 | 844.25±90.98 | 133.482 |
|  | Program A | 3 | 14±3.78 | 91.99±25.64 | 224.57±14.03 | 316.56±33.88 | - |
|  | Program B |  | 7±1.87 | 48.81±11.89 | 795.44±86.62 | 844.25±90.98 | 82.971 |
|  | Program A | 4 | 17±5.05 | 117.79±34.29 | 229.99±15.51 | 347.78±39.67 | - |
|  | Program B |  | 7±1.87 | 48.81±11.89 | 795.44±86.62 | 844.25±90.98 | 48.866 |
|  | Program A | 7 | 41±12.74 | 278.29±83.57 | 259.70±93.55 | 537.99±105.56 |  |
|  | Program B |  | 7±1.87 | 48.81±11.89 | 795.44±86.62 | 844.25±90.98 | 9.008 |
|  | Program A | 10 | 101±327.33 | 605.20±2,530.45 | 267.48±218.36 | 872.68±2,276.18 |  |
|  | Program B |  | 7±1.87 | 48.81±11.89 | 795.44±86.62 | 844.25±90.98 | cost-saving |

*ICERs (incremental cost-effectiveness ratios): Program B comparing to Program A.

**Table A.8 The impact of quarantine probability in scenario I (US$1,000)**

| Probability  (%) | Strategy | Number of cases | Cost | | | ICERs |
| --- | --- | --- | --- | --- | --- | --- |
|  |  |  | Cost of cases | Cost of measures | Total cost |  |
| 25 | Program A | 2±1.79 | 11.81±12.15 | 204.93±51.49 | 216.74±60.47 | - |
|  | Program B | 1±0.48 | 8.55±3.29 | 712.60±60.02 | 721.15±60.79 | 1,050.856 |
| 50 | Program A | 1±0.57 | 8.35±3.84 | 178.92±26.14 | 187.27±27.27 | dominating |
|  | Program B | 1±0.48 | 8.55±3.29 | 712.60±60.02 | 721.15±60.79 | - |
| 75 | Program A | 1±0.58 | 8.96±3.96 | 178.05±13.76 | 187.02±15.38 | dominating |
|  | Program B | 1±0.48 | 8.55±3.29 | 712.60±60.02 | 721.15±60.79 | - |
| 100 | Program A | 1±0.47 | 8.35±3.18 | 169.08±25.67 | 177.43±26.86 | dominating |
|  | Program B | 1±0.48 | 8.55±3.29 | 712.60±60.02 | 721.15±60.79 | - |

* Program A: personal protection and isolation-and-quarantine; Program B: personal protection and community containment; ICERs (incremental cost-effectiveness ratios): Program B comparing to Program A.

**Table A.9 The impact of quarantine probability in scenario II (US$1,000)**

| Probability  (%) | Strategy | Number of cases | Cost | | | ICERs |
| --- | --- | --- | --- | --- | --- | --- |
|  |  |  | Cost of cases | Cost of measures | Total cost |  |
| 25 | Program A | 34±163.09 | 230.24±1,107.26 | 308.92±171.89 | 539.16±1,253.93 | - |
|  | Program B | 7±1.87 | 48.81±11.89 | 795.44±86.62 | 844.25±90.98 | 11.417 |
| 50 | Program A | 8±2.47 | 51.73±16.76 | 214.98±22.42 | 266.72±35.75 | - |
|  | Program B | 7±1.87 | 48.81±11.89 | 795.44±86.62 | 844.25±90.98 | 1,343.109 |
| 75 | Program A | 8±2.06 | 50.65±14.00 | 199.24±14.18 | 249.88±22.94 | - |
|  | Program B | 7±1.87 | 48.81±11.89 | 795.44±86.62 | 844.25±90.98 | 2,201.374 |
| 100 | Program A | 7±2.07 | 49.15±14.05 | 189.54±13.62 | 238.70±22.76 | - |
|  | Program B | 7±1.87 | 48.81±11.89 | 795.44±86.62 | 844.25±90.98 | 12,111.151 |

* Program A: personal protection and isolation-and-quarantine; Program B: personal protection and community containment; ICERs (incremental cost-effectiveness ratios): Program B comparing to Program A.

**Table A.10 Threshold analysis of cost-effectiveness of quarantine probability (US$1,000)**

| Strategy | Probability  (%) | Number of cases | Total cost | ICERs |
| --- | --- | --- | --- | --- |
|  |  |  |  |  |
| Program A | 20 | 198±515.79 | 1,766.10±3,811.61 | cost-saving |
|  | 22 | 65±277.63 | 761.49±2,122.08 | 1.440 |
|  | 23 | 60±235.60 | 743.57±1,804.56 | 1.944 |
|  | 24 | 57±234.47 | 704.58±1,813.10 | 2.814 |
|  | 25 | 34±163.09 | 539.16±1,253.93 | 11.417 |
|  | 26 | 30±153.01 | 499.73±1,182.35 | 15.157 |
|  | 27 | 27±141.87 | 480.56±1,095.50 | 18.099 |
|  | 28 | 20±105.23 | 412.37±828.17 | 33.900 |
|  | 29 | 17±89.85 | 384.26±713.76 | 46.015 |
|  | 30 | 16±78.36 | 371.26±613.01 | 56.021 |
|  | 35 | 8±8.48 | 300.57±69.22 | 421.457 |
| Program B | | 7±1.87 | 844.25±90.98 | - |

Program A: personal protection and isolation-and-quarantine; Program B: personal protection and community containment; ICERs (incremental cost-effectiveness ratios): Program B comparing to Program A.

**Table A.11 Comparisons of impact of various quarantine delay time in scenario I (US$1,000)**

| Scenario | Delay days | Strategy | Number of cases | Cost | | | ICERs |
| --- | --- | --- | --- | --- | --- | --- | --- |
|  |  |  |  | Cost of cases | Cost of measures | Total cost |  |
| I | 0 | Program A | 1±0.39 | 8.01±2.62 | 159.62±19.69 | 167.63±20.53 | dominating |
|  | - | Program B | 1±0.48 | 8.55±3.29 | 712.60±60.02 | 721.15±60.79 | - |
|  | 2 | Program A | 1±0.47 | 8.35±3.18 | 169.08±25.67 | 177.43±26.86 | dominating |
|  | - | Program B | 1±0.48 | 8.55±3.29 | 712.60±60.02 | 721.15±60.79 | - |
|  | 3 | Program A | 1±0.55 | 9.03±3.74 | 178.76±17.11 | 187.78±18.49 | - |
|  | - | Program B | 1±0.48 | 8.55±3.29 | 712.60±60.02 | 721.15±60.79 | 7,619.61 |
|  | 4 | Program A | 1±0.74 | 9.03±5.02 | 185.54±21.44 | 194.57±24.15 | - |
|  | - | Program B | 1±0.48 | 8.55±3.29 | 712.60±60.02 | 721.15±60.79 | 7,522.64 |
|  | 5 | Program A | 1±1.05 | 9.03±7.16 | 194.90±29.73 | 203.93±35.77 | - |
|  | - | Program B | 1±0.48 | 8.55±3.29 | 712.60±60.02 | 721.15±60.79 | 7,388.98 |
|  | 6 | Program A | 37±246.31 | 249.64±1,672.22 | 225.24±127.55 | 474.87±1,791.41 | - |
|  | - | Program B | 1±0.48 | 8.55±3.29 | 712.60±60.02 | 721.15±60.79 | 6.935 |
|  | 7 | Program A | 118±460.65 | 801.82±3,127.45 | 277.95±235.28 | 1,079.77±3,356.62 |  |
|  | - | Program B | 1±0.48 | 8.55±3.29 | 712.60±60.02 | 721.15±60.79 | cost-saving |
|  | 10 | Program A | 120±476.29 | 817.55±3,233.60 | 253.43±165.18 | 1,070.99±3,386.10 |  |
|  | - | Program B | 1±0.48 | 8.55±3.29 | 712.60±60.02 | 721.15±60.79 | cost-saving |
|  | 14 | Program A | 161±546.92 | 1,091.02±3713.13 | 268.24±158.49 | 1,359.26±3,861.89 |  |
|  | - | Program B | 1±0.48 | 8.55±3.29 | 712.60±60.02 | 721.15±60.79 | cost-saving |
|  | 21 | Program A | 193±594.03 | 1,309.92±4,032.96 | 295.51±157.53 | 1,605.43±4,172.44 |  |
|  | - | Program B | 1±0.48 | 8.55±3.29 | 712.60±60.02 | 721.15±60.79 | cost-saving |

* Program A: personal protection and isolation-and-quarantine; Program B: personal protection and community containment; ICERs (incremental cost-effectiveness ratios): Program B comparing to Program A.

**Table A.12 Comparisons of impact of various quarantine delay time in scenario II (US$1,000)**

| Scenario | Delay days | Strategy | Number of cases | Cost | | | ICERs |
| --- | --- | --- | --- | --- | --- | --- | --- |
|  |  |  |  | Cost of cases | Cost of measures | Total cost |  |
| II | 0 | Program A | 7±1.38 | 46..23±9.39 | 176.82±13.03 | 223.06±18.35 | dominating |
|  | - | Program B | 7±1.87 | 48.81±11.89 | 795.44±86.62 | 844.25±90.98 | - |
|  | 2 | Program A | 7±2.07 | 49.15±14.05 | 189.54±13.62 | 238.70±22.76 | - |
|  | - | Program B | 7±1.87 | 48.81±11.89 | 795.44±86.62 | 844.25±90.98 | 12,111.151 |
|  | 3 | Program A | 7±2.40 | 49.90±16.29 | 201.42±18.29 | 251.32±31.87 | - |
|  | - | Program B | 7±1.87 | 48.81±11.89 | 795.44±86.62 | 844.25±90.98 | 3,705.856 |
|  | 4 | Program A | 9±4.58 | 58.39±31.03 | 223.59±30.78 | 281.98±59.02 | - |
|  | - | Program B | 7±1.87 | 48.81±11.89 | 795.44±86.62 | 844.25±90.98 | 398.774 |
|  | 5 | Program A | 50±242.30 | 340.27±1645.00 | 276.25±147.82 | 616.52±1,772.29 | - |
|  | - | Program B | 7±1.87 | 48.81±11.89 | 795.44±86.62 | 844.25±90.98 | 5.305 |
|  | 6 | Program A | 553±808.45 | 3,752.02±5,488.71 | 499.96±347.23 | 4,251.98±5,815.69 | - |
|  | - | Program B | 7±1.87 | 48.81±11.89 | 795.44±86.62 | 844.25±90.98 | cost-saving |
|  | 7 | Program A | 859±963.83 | 5,831.57±6,543.64 | 602.01±347.65 | 6,433.57±6,863.67 |  |
|  | - | Program B | 7±1.87 | 48.81±11.89 | 795.44±86.62 | 844.25±90.98 | cost-saving |
|  | 10 | Program A | 1,589±812.78 | 10,788.69±5,518.09 | 627.91±214.66 | 11416.60±5,712.96 |  |
|  | - | Program B | 7±1.87 | 48.81±11.89 | 795.44±86.62 | 844.25±90.98 | cost-saving |
|  | 14 | Program A | 1,795±612.21 | 12,186.92±4,156.39 | 669.12±156.33 | 12,856.05±4,286.51 |  |
|  | - | Program B | 7±1.87 | 48.81±11.89 | 795.44±86.62 | 844.25±90.98 | cost-saving |
|  | 21 | Program A | 1,824±574.13 | 12,383.47±3,897.88 | 629.86±154.06 | 13,013.33±3,990.14 |  |
|  | - | Program B | 7±1.87 | 48.81±11.89 | 795.44±86.62 | 844.25±90.98 | cost-saving |

* Program A: personal protection and isolation-and-quarantine; Program B: personal protection and community containment; ICERs (incremental cost-effectiveness ratios): Program B comparing to Program A.

**Table A.13 Comparisons of impact of cost of patient in scenario I (US$1,000)**

| Cost of patient | Strategy | Cost | | | ICERs |
| --- | --- | --- | --- | --- | --- |
|  |  | Cost of cases | Cost of measures | Total cost |  |
| 2,900 | No intervention | 5,415.39±2,284.77 | 0 | 5,415.39±2,284.77 | - |
|  | Isolation-and-quarantine | 4.91±3.46 | 1.97±1.55 | 6.88±4.57 | cost-saving |
|  | Program A | 4.34±2.15 | 170.07±22.89 | 174.40±23.81 | cost-saving |
|  | Program B | 4.02±1.55 | 712.60±60.02 | 716.62±60.36 | cost-saving |
| 6,500 | No intervention | 11,528.37±4,863.86 | 0 | 11,528.37±4,863.86 | - |
|  | Isolation-and-quarantine | 10.46±7.38 | 1.97±1.55 | 12.43±8.40 | cost-saving |
|  | Program A | 9.23±4.58 | 170.07±22.89 | 179.30±25.04 | cost-saving |
|  | Program B | 8.55±3.29 | 712.60±60.02 | 721.15±60.79 | cost-saving |
| 10,000 | No intervention | 17,471.55±7,371.31 | 0 | 17,471.55±7,371.31 | - |
|  | Isolation-and-quarantine | 15.85±11.18 | 1.97±1.55 | 17.82±12.17 | cost-saving |
|  | Program A | 13.99±6.94 | 170.07±22.89 | 184.06±26.39 | cost-saving |
|  | Program B | 12.96±4.98 | 712.60±60.02 | 725.56±61.25 | cost-saving |

* Program A: personal protection and isolation-and-quarantine; Program B: personal protection and community containment. ICERs (incremental cost-effectiveness ratios): comparing to the no intervention.

**Table A.14 Comparisons of impact of cost of patient in scenario II (US$1,000)**

| Cost of patient | Strategy | Cost | | | ICERs |
| --- | --- | --- | --- | --- | --- |
|  |  | Cost of cases | Cost of measures | Total cost |  |
| 2,900 | No intervention | 6,372.08±6.37 | 0 | 6,372.08±6.37 | - |
|  | Isolation-and-quarantine | 24.59±7.20 | 6.21±2.61 | 30.80±8.62 | cost-saving |
|  | Program A | 23.09±6.60 | 189.54±13.62 | 212.63±17.10 | cost-saving |
|  | Program B | 22.93±5.58 | 795.44±86.62 | 818.37±88.50 | cost-saving |
| 6,500 | No intervention | 13,564.99±13.56 | 0 | 13,564.99±13.56 | - |
|  | Isolation-and-quarantine | 52.34±15.33 | 6.21±2.61 | 58.56±16.58 | cost-saving |
|  | Program A | 49.15±14.05 | 189.54±13.62 | 238.70±22.76 | cost-saving |
|  | Program B | 48.81±11.89 | 795.44±86.62 | 844.25±90.98 | cost-saving |
| 10,000 | No intervention | 20,558.10±20.55 | 0 | 20,558.10±20.55 | - |
|  | Isolation-and-quarantine | 79.33±23.23 | 6.21±2.61 | 85.54±16.58 | cost-saving |
|  | Program A | 74.49±21.30 | 189.54±13.62 | 264.04±29.05 | cost-saving |
|  | Program B | 74.98±18.01 | 795.44±86.62 | 869.42±93.84 | cost-saving |

* Program A: personal protection and isolation-and-quarantine; Program B: personal protection and community containment; ICERs (incremental cost-effectiveness ratios): comparing to the no intervention.

**Table A.15 The impact of transmission constant and quarantine probability in scenario I (US$1,000)**

| Transmission constant | Probability (%) | Number of cases | Total cost | ICERs |
| --- | --- | --- | --- | --- |
| 0.25 | 25 | 1±0.36 | 202.83±36.46 | dominating |
|  | 50 | 1±0.31 | 185.31±28.94 | dominating |
|  | 75 | 1±0.20 | 175.48±24.68 | dominating |
|  | 100 | 1±0.22 | 172.80±23.82 | dominating |
| 0.50 | 25 | 1±1.18 | 203.74±57.35 | dominating |
|  | 50 | 1±0.44 | 188.02±24.76 | dominating |
|  | 75 | 1±0.35 | 179.13±18.68 | dominating |
|  | 100 | 1±0.37 | 177.07±19.22 | dominating |
| 0.75 | 25 | 1±0.40 | 207.38±36.28 | 12,493.009 |
|  | 50 | 1±0.48 | 187.80±26.04 | dominating |
|  | 75 | 1±0.39 | 183.58±20.08 | dominating |
|  | 100 | 1±0.45 | 175.06±22.86 | dominating |
| 1 | 25 | 2±1.79 | 216.74±60.47 | 1,050.856 |
|  | 50 | 1±0.57 | 187.27±27.27 | dominating |
|  | 75 | 1±0.58 | 187.02±15.38 | dominating |
|  | 100 | 1±0.47 | 177.43±26.86 | dominating |
| 2 | 25 | 2±2.90 | 218.75±73.23 | 982.571 |
|  | 50 | 1±0.78 | 193.22±27.45 | dominating |
|  | 75 | 1±0.75 | 183.09±23.04 | dominating |
|  | 100 | 1±0.69 | 178.47±25.00 | dominating |

* Program A: personal protection and isolation-and-quarantine; ICERs (incremental cost-effectiveness ratios): Program B comparing to Program A.

**Table A.16 The impact of transmission constant and quarantine probability in scenario II (US$1,000)**

| Transmission constant | Probability (%) | Number of cases | Total cost | ICERs |
| --- | --- | --- | --- | --- |
| 0.25 | 25 | 5±1.35 | 280.03±42.24 | 5,716.38 |
|  | 50 | 5±0.93 | 239.76±20.91 | dominating |
|  | 75 | 5±0.92 | 224.72±13.75 | dominating |
|  | 100 | 5±0.95 | 219.45±16.61 | dominating |
| 0.50 | 25 | 6±2.84 | 299.98±59.37 | 1,270.04 |
|  | 50 | 6±1.44 | 246.02±26.17 | dominating |
|  | 75 | 6±1.34 | 232.11±17.27 | dominating |
|  | 100 | 6±1.52 | 228.02±21.18 | dominating |
| 0.75 | 25 | 10±7.99 | 346.63±131.94 | 151.227 |
|  | 50 | 7±2.03 | 255.68±27.07 | 4,787.19 |
|  | 75 | 6±1.52 | 236.56±20.31 | dominating |
|  | 100 | 6±1.60 | 232.37±25.70 | dominating |
| 1 | 25 | 34±163.09 | 539.16±1,253.93 | 11.417 |
|  | 50 | 8±2.47 | 266.72±35.75 | 1,343.109 |
|  | 75 | 8±2.06 | 249.88±22.94 | 2,201.374 |
|  | 100 | 7±2.07 | 238.70±22.76 | 12,111.151 |
| 2 | 25 | 430.19±634.81 | 3,504.82±4,721.05 | -9.149 |
|  | 50 | 10±4.70 | 292.12±58.52 | 438.499 |
|  | 75 | 9±2.93 | 257.37±31.88 | 2,763.644 |
|  | 100 | 9±2.29 | 249.80±24.74 | 2,263.600 |

* Program A: personal protection and isolation-and-quarantine; ICERs (incremental cost-effectiveness ratios): Program B comparing to Program A.

**Table A.17 The impact of isolation delay time and quarantine probability in scenario I (US$1,000)**

| Probability (%) | Isolation delay day | Number of cases | Total cost | ICERs |
| --- | --- | --- | --- | --- |
| 25 | 1 | 3±5.40 | 277.75±86.26 | 203.398 |
|  | 2 | 5±7.90 | 300.09±109.50 | 128.374 |
|  | 3 | 6±22.91 | 311.64±245.28 | 81.252 |
|  | 4 | 8±22.47 | 333.16±249.46 | 60.154 |
| 50 | 1 | 2±0.92 | 207.07±28.34 | 1,195.554 |
|  | 2 | 2±1.46 | 231.03±33.47 | 426.192 |
|  | 3 | 2±1.48 | 230.85±31.34 | 405.211 |
|  | 4 | 3±2.00 | 242.30±36.20 | 280.032 |
| 75 | 1 | 2±0.93 | 200.80±17.08 | 1,626.117 |
|  | 2 | 2±1.16 | 210.89±23.42 | 671.404 |
|  | 3 | 2±1.32 | 214.34±26.72 | 589.314 |
|  | 4 | 2±1.50 | 225.24±23.01 | 390.482 |
| 100 | 1 | 1±0.67 | 191.13±20.72 | 2,409.204 |
|  | 2 | 2±1.17 | 201.08±21.31 | 702.805 |
|  | 3 | 2±1.03 | 209.70±17.30 | 549.946 |
|  | 4 | 2±1.27 | 210.20±23.39 | 537.850 |

* Program A: personal protection and isolation-and-quarantine; ICERs (incremental cost-effectiveness ratios): Program B comparing to Program A.

**Table A.18 The impact of isolation delay time and quarantine probability in scenario II (US$1,000)**

| Probability (%) | Isolation delay day | Number of cases | Total cost | ICERs |
| --- | --- | --- | --- | --- |
| 25 | 1 | 37±46.68 | 656.67±443.61 | 6.209 |
|  | 2 | 42±41.95 | 712.99±428.76 | 3.760 |
|  | 3 | 43±44.59 | 706.42±422.23 | 3.817 |
|  | 4 | 62±55.44 | 838.40±462.51 | 0.108 |
| 50 | 1 | 15±6.00 | 360.46±70.22 | 61.946 |
|  | 2 | 16±6.47 | 368.30±62.16 | 54.024 |
|  | 3 | 22±6.83 | 427.36±71.62 | 27.961 |
|  | 4 | 24±8.10 | 453.85±96.01 | 23.294 |
| 75 | 1 | 11±3.72 | 298.13±37.88 | 129.722 |
|  | 2 | 13±3.63 | 309.83±41.13 | 96.992 |
|  | 3 | 17±4.42 | 354.85±40.38 | 50.926 |
|  | 4 | 17±5.56 | 359.40±49.86 | 49.424 |
| 100 | 1 | 10±2.86 | 272.86±29.31 | 196.356 |
|  | 2 | 11±3.38 | 288.97±31.98 | 133.482 |
|  | 3 | 14±3.78 | 316.56±33.88 | 82.971 |
|  | 4 | 17±5.05 | 347.78±39.67 | 48.866 |

* Program A: personal protection and isolation-and-quarantine; ICERs (incremental cost-effectiveness ratios): Program B comparing to Program A.

**Table A.19 The impact of quarantine delay time and probability in scenario I (US$1,000)**

| Probability (%) | Quarantine delay day | Number of cases | Total cost | ICERs |
| --- | --- | --- | --- | --- |
| 25 | 2 | 2±1.79 | 216.74±60.47 | 1,050.856 |
|  | 3 | 17±159.37 | 344.17±1,228.73 | 23.547 |
|  | 4 | 20±183.24 | 363.57±1,322.08 | 19.030 |
|  | 5 | 79±380.30 | 786.64±2,711.09 | -0.844 |
| 50 | 2 | 1±0.57 | 187.27±27.27 | dominating |
|  | 3 | 1±1.19 | 207.46±39.92 | 1,027.382 |
|  | 4 | 2±1.30 | 214.31±44.04 | 1,206.766 |
|  | 5 | 19±179.07 | 341.05±1,324.18 | 21.082 |
| 75 | 2 | 1±0.58 | 187.02±15.38 | dominating |
|  | 3 | 1±0.64 | 193.33±30.30 | 3,298.886 |
|  | 4 | 2±1.13 | 204.50±35.05 | 1,519.565 |
|  | 5 | 2±1.26 | 208.50±44.11 | 1,424.027 |
| 100 | 2 | 1±0.47 | 177.43±26.86 | dominating |
|  | 3 | 1±0.67 | 184.43±30.03 | 4,472.730 |
|  | 4 | 2±0.58 | 189.00±27.04 | 3,325.972 |
|  | 5 | 2±1.02 | 204.24±28.84 | 2,153.798 |

* Program A: personal protection and isolation-and-quarantine; ICERs (incremental cost-effectiveness ratios): Program B comparing to Program A.

**Table A.20 The impact of quarantine delay time and probability in scenario II (US$1,000)**

| Probability (%) | Quarantine delay day | Number of cases | Total cost | ICERs |
| --- | --- | --- | --- | --- |
| 25 | 2 | 34±163.09 | 539.16±1,253.93 | 11.417 |
|  | 3 | 291±626.100 | 2,423.30±4,580.65 | cost-saving |
|  | 4 | 691±884.28 | 6,063.87±6,728.78 | cost-saving |
|  | 5 | 811±949.13 | 9,435.42±6,589.48 | cost-saving |
| 50 | 2 | 8±2.47 | 266.72±35.75 | 1,343.109 |
|  | 3 | 8±3.40 | 280.40±48.18 | 924.354 |
|  | 4 | 52±226.34 | 641.44±1,694.07 | 4.521 |
|  | 5 | 302±652.39 | 2,460.63±4,712.94 | cost-saving |
| 75 | 2 | 8±2.06 | 249.88±22.94 | 2,201.374 |
|  | 3 | 8±2.55 | 263.15±33.94 | 1,263.264 |
|  | 4 | 9±9.33 | 295.98±94.45 | 301.249 |
|  | 5 | 154±444.60 | 1,393.74±3,247.68 | cost-saving |
| 100 | 2 | 7±2.07 | 238.70±22.76 | dominating |
|  | 3 | 7±2.40 | 251.32±31.87 | 3,705.856 |
|  | 4 | 9±4.57 | 281.98±59.02 | 398.774 |
|  | 5 | 50±242.30 | 616.52±1,775.29 | 5.305 |

* Program A: personal protection and isolation-and-quarantine; ICERs (incremental cost-effectiveness ratios): Program B comparing to Program A.

**Table A.21 The impact of cost of quarantine and quarantine probability (US$1,000)**

| Scenario | Cost of quarantine | Quarantine probability | Program A | Program B | ICERs |
| --- | --- | --- | --- | --- | --- |
|  |  |  | Total cost | Total cost |  |
| I | 50 | 25 | 216.74±60.47 | 721.15±60.79 | 1,050.856 |
|  |  | 50 | 187.27±27.27 | 721.15±60.79 | dominating |
|  |  | 75 | 187.02±15.38 | 721.15±60.79 | dominating |
|  |  | 100 | 177.43±26.86 | 721.15±60.79 | dominating |
|  | 100 | 25 | 219.26±63.84 | 722.59±61.18 | 1,048.613 |
|  |  | 50 | 189.00±27.97 | 722.59±61.18 | dominating |
|  |  | 75 | 188.67±15.99 | 722.59±61.18 | dominating |
|  |  | 100 | 178.91±27.46 | 722.59±61.18 | dominating |
|  | 150 | 25 | 221.77±67.27 | 724.03±61.59 | 1,046.371 |
|  |  | 50 | 190.73±28.69 | 724.03±61.59 | dominating |
|  |  | 75 | 190.33±16.65 | 724.03±61.59 | dominating |
|  |  | 100 | 180.38±28.10 | 724.03±61.59 | dominating |
| II | 50 | 25 | 539.16±1,253.93 | 844.25±90.98 | 11.417 |
|  |  | 50 | 266.72±35.75 | 844.25±90.98 | 1,343.109 |
|  |  | 75 | 249.88±22.94 | 844.25±90.98 | 2,201.375 |
|  |  | 100 | 238.70±22.76 | 844.25±90.98 | 1,2111.152 |
|  | 100 | 25 | 560.75±1,301.95 | 849.56±91.51 | 10.807 |
|  |  | 50 | 274.19±38.76 | 849.56±91.51 | 1,338.072 |
|  |  | 75 | 256.12±24.42 | 849.56±91.51 | 2,197.925 |
|  |  | 100 | 243.81±23.43 | 849.56±91.51 | 12,114.862 |
|  | 150 | 25 | 582.34±1,350.18 | 854.86±92.08 | 10.198 |
|  |  | 50 | 281.66±41.87 | 854.86±92.08 | 1,333.035 |
|  |  | 75 | 262.35±26.07 | 854.86±92.08 | 2,194.475 |
|  |  | 100 | 248.93±24.23 | 854.86±92.08 | 12,118.572 |

*Program A: personal protection and isolation-and-quarantine; ICERs (incremental cost-effectiveness ratios): Program B comparing to Program A, cost of quarantine referred to the cost of quarantine per close contact per day.

**Table A.22 The impact of cost of community containment and quarantine probability (US$1,000)**

| Scenario | Weight | Quarantine probability | Program A | Program B | ICERs |
| --- | --- | --- | --- | --- | --- |
|  |  |  | Total cost | Total cost |  |
| I | 0.4 | 25 | 216.74±60.47 | 446.90±38.15 | 479.500 |
|  |  | 50 | 187.27±27.27 | 446.90±38.15 | dominating |
|  |  | 75 | 187.02±15.38 | 446.90±38.15 | dominating |
|  |  | 100 | 177.43±26.86 | 446.90±38.15 | dominating |
|  | 0.6 | 25 | 216.74±60.47 | 584.75±49.66 | 766.675 |
|  |  | 50 | 187.27±27.27 | 584.75±49.66 | dominating |
|  |  | 75 | 187.02±15.38 | 584.75±49.66 | dominating |
|  |  | 100 | 177.43±26.86 | 584.75±49.66 | dominating |
|  | 0.8 | 25 | 216.74±60.47 | 721.15±60.79 | 1,050.856 |
|  |  | 50 | 187.27±27.27 | 721.15±60.79 | dominating |
|  |  | 75 | 187.02±15.38 | 721.15±60.79 | dominating |
|  |  | 100 | 177.43±26.86 | 721.15±60.79 | dominating |
|  | 1 | 25 | 216.74±60.47 | 860.43±72.71 | 1,341.023 |
|  |  | 50 | 187.27±27.27 | 860.43±72.71 | dominating |
|  |  | 75 | 187.02±15.38 | 860.43±72.71 | dominating |
|  |  | 100 | 177.43±26.86 | 860.43±72.71 | dominating |
| II | 0.4 | 25 | 539.16±1,253.93 | 543.60±58.64 | 0.166 |
|  |  | 50 | 266.72±35.75 | 543.60±58.64 | 643.919 |
|  |  | 75 | 249.88±22.94 | 543.60±58.64 | 1,087.849 |
|  |  | 100 | 238.70±22.76 | 543.60±58.64 | 6,098.115 |
|  | 0.6 | 25 | 539.16±1,253.93 | 656.58±75.02 | 5.891 |
|  |  | 50 | 266.72±35.75 | 656.58±75.02 | 999.681 |
|  |  | 75 | 249.88±22.94 | 656.58±75.02 | 1,654.433 |
|  |  | 100 | 238.70±22.76 | 656.58±75.02 | 9,157.668 |
|  | 0.8 | 25 | 539.16±1,253.93 | 844.25±90.98 | 11.417 |
|  |  | 50 | 266.72±35.75 | 844.25±90.98 | 1,343.109 |
|  |  | 75 | 249.88±22.94 | 844.25±90.98 | 2,201.375 |
|  |  | 100 | 238.70±22.76 | 844.25±90.98 | 12,111.152 |
|  | 1 | 25 | 539.16±1,253.93 | 1,002.54±108.06 | 17.340 |
|  |  | 50 | 266.72±35.75 | 1,002.54±108.06 | 1,711.205 |
|  |  | 75 | 249.88±22.94 | 1,002.54±108.06 | 2,787.601 |
|  |  | 100 | 238.70±22.76 | 1,002.54±108.06 | 15,276.776 |

* Program A: personal protection and isolation-and-quarantine; ICERs (incremental cost-effectiveness ratios): Program B comparing to Program A; weight: the calculation weight of cost of community containment.

**Table A.23 The impact of cost of quarantine and quarantine delay time (S$1,000)**

| Scenario | Cost of quarantine | Quarantine delay day | Program A | Program B | ICERs |
| --- | --- | --- | --- | --- | --- |
|  |  |  | Total cost | Total cost |  |
| I | 50 | 3 | 188.02±18.67 | 721.15±60.79 | 17,770.982 |
|  |  | 4 | 194.64±23.57 | 721.15±60.79 | 7,521.612 |
|  |  | 5 | 199.02±39.68 | 721.15±60.79 | 4,746.688 |
|  |  | 6 | 456.74±1,779.29 | 721.15±60.79 | 7.579 |
|  | 100 | 3 | 189.94±19.44 | 722.59±61.18 | 17,755.065 |
|  |  | 4 | 193.37±24.67 | 722.59±61.18 | 7,517.476 |
|  |  | 5 | 201.042±40.45 | 722.59±61.18 | 4,741.342 |
|  |  | 6 | 462.37±1,802.66 | 722.59±61.18 | 7.458 |
|  | 150 | 3 | 191.85±20.28 | 724.03±61.59 | 17,739.149 |
|  |  | 4 | 198.09±25.81 | 724.03±61.59 | 7,513.340 |
|  |  | 5 | 203.07±42.26 | 724.03±61.59 | 4,735.997 |
|  |  | 6 | 468.00±1,826.03 | 724.03±61.59 | 7.338 |
| II | 50 | 3 | 251.32±31.87 | 844.25±90.98 | 3,705.856 |
|  |  | 4 | 281.98±59.02 | 844.25±90.98 | 398.774 |
|  |  | 5 | 616.52±1,775.29 | 844.25±90.98 | 5.305 |
|  |  | 6 | 4,251.98±5,815.69 | 844.25±90.98 | -6.247 |
|  | 100 | 3 | 257.87±34.23 | 849.56±91.51 | 3,698.034 |
|  |  | 4 | 291.00±64.34 | 849.56±91.51 | 396.140 |
|  |  | 5 | 636.11±1,813.69 | 849.56±91.51 | 4.972 |
|  |  | 6 | 4,311.08±5,819.93 | 849.56±91.51 | cost-saving |
|  | 150 | 3 | 264.43±36.74 | 854.86±92.08 | 3,690.212 |
|  |  | 4 | 300.02±69.75 | 854.86±92.08 | 393.506 |
|  |  | 5 | 655.69±1,852.20 | 854.86±92.08 | 4.639 |
|  |  | 6 | 4,370.17±5,944.25 | 854.86±92.08 | cost-saving |

* Program A: personal protection and isolation-and-quarantine; ICERs (incremental cost-effectiveness ratios): Program B comparing to Program A, cost of quarantine referred to the cost of quarantine per close contact per day.

**Table A.24 The impact of cost of community containment and quarantine delay time (US$1,000)**

| Scenario | Weight | Quarantine delay day | Program A | Program B | ICERs |
| --- | --- | --- | --- | --- | --- |
|  |  |  | Total cost | Total cost |  |
| I | 0.4 | 3 | 188.02±18.67 | 446.90±38.15 | 8,675.871 |
|  |  | 4 | 194.64±23.57 | 446.90±38.15 | 3,623.707 |
|  |  | 5 | 199.02±39.68 | 446.90±38.15 | 2,266.203 |
|  |  | 6 | 456.74±1,779.29 | 446.90±38.15 | cost-saving |
|  | 0.6 | 3 | 188.02±18.67 | 584.75±49.66 | 13,224.073 |
|  |  | 4 | 194.64±23.57 | 584.75±49.66 | 5,572.936 |
|  |  | 5 | 199.02±39.68 | 584.75±49.66 | 3,506.622 |
|  |  | 6 | 456.74±1,779.29 | 584.75±49.66 | 3.669 |
|  | 0.8 | 3 | 188.02±18.67 | 721.15±60.79 | 17,770.982 |
|  |  | 4 | 194.64±23.57 | 721.15±60.79 | 7,521.612 |
|  |  | 5 | 199.02±39.68 | 721.15±60.79 | 4,746.688 |
|  |  | 6 | 456.74±1,779.29 | 721.15±60.79 | 7.579 |
|  | 1 | 3 | 188.02±18.67 | 860.43±72.71 | 22,413.658 |
|  |  | 4 | 194.64±23.57 | 860.43±72.71 | 9,511.330 |
|  |  | 5 | 199.02±39.68 | 860.43±72.71 | 6,012.872 |
|  |  | 6 | 456.74±1,779.29 | 860.43±72.71 | 11.571 |
| II | 0.4 | 3 | 251.32±31.87 | 543.60±58.64 | 1,826.782 |
|  |  | 4 | 281.98±59.02 | 543.60±58.64 | 185.546 |
|  |  | 5 | 616.52±1,775.29 | 543.60±58.64 | cost-saving |
|  |  | 6 | 4,251.98±5,815.69 | 543.60±58.64 | cost-saving |
|  | 0.6 | 3 | 251.32±31.87 | 656.58±75.02 | 2,782.892 |
|  |  | 4 | 281.98±59.02 | 656.58±75.02 | 294.041 |
|  |  | 5 | 616.52±1,775.29 | 656.58±75.02 | 1.865 |
|  |  | 6 | 4,251.98±5,815.69 | 656.58±75.02 | cost-saving |
|  | 0.8 | 3 | 251.32±31.87 | 844.25±90.98 | 3,705.856 |
|  |  | 4 | 281.98±59.02 | 844.25±90.98 | 398.774 |
|  |  | 5 | 616.52±1,775.29 | 844.25±90.98 | 5.305 |
|  |  | 6 | 4,251.98±5,815.69 | 844.25±90.98 | cost-saving |
|  | 1 | 3 | 251.32±31.87 | 1,002.54±108.06 | 4,695.113 |
|  |  | 4 | 281.98±59.02 | 1,002.54±108.06 | 511.031 |
|  |  | 5 | 616.52±1,775.29 | 1,002.54±108.06 | 8.992 |
|  |  | 6 | 4,251.98±5,815.69 | 1,002.54±108.06 | cost-saving |

* Program A: personal protection and isolation-and-quarantine; ICERs (incremental cost-effectiveness ratios): Program B comparing to Program A; weight: the calculation weight of cost of community containment.
